# Availability and Access to Palliative Sedation Medications in Ecuador: A National Survey of Palliative Care Physicians

**DOI:** 10.3390/ijerph23070901

**Published:** 2026-07-14

**Authors:** Patricia Bonilla-Sierra, Luz Damaris Villalta-Tandazo

**Affiliations:** 1 Faculty of Health Sciences, Universidad Técnica Particular de Loja (UTPL), Loja 110107, Ecuador; pbonilla65@utpl.edu.ec; 2 Department of Health Sciences, Universidad Técnica Particular de Loja (UTPL), Loja 110107, Ecuador

**Keywords:** palliative care, sedation, drug accessibility, Ecuador, medication availability, end-of-life care, health system barriers, public health

## Abstract

**Highlights:**

**Public health relevance—How does this work relate to a public health issue?**

- This study frames access to palliative sedation medications as a public health issue in Ecuador, showing how irregular availability can limit symptom control and affect end-of-life care.

**Public health significance—Why is this work of significance to public health?**

- This study provides national evidence on systemic barriers to palliative sedation medication access, including regulatory restrictions, administrative burden, distribution problems, and institutional disparities.

**Public health implications—What are the key implications or messages for practitioners, policy makers, and/or researchers in public health?**

- The findings support the need for clearer policies, reliable supply systems, professional training, and coordinated action among health authorities, institutions, pharmacies, and scientific associations to improve equitable access to palliative sedation.

**Abstract:**

Palliative sedation is a key intervention to relieve refractory suffering at the end of life; however, its implementation depends on access to essential medications. This study aimed to evaluate the availability and access to palliative sedation medications and to identify associated barriers among physicians affiliated with the Ecuadorian Association of Palliative Care (ASECUP). A quantitative, cross-sectional study was conducted using a structured, validated questionnaire administered to 82 eligible ASECUP-affiliated physicians. Descriptive and bivariate analyses were performed to assess associations between medication availability and institutional variables. Midazolam was the most frequently used medication (82.0%); however, availability was inconsistent, with only 54.9% reporting constant access. The main barriers identified were regulatory restrictions (53.7%), distribution problems (36.6%), and the absence of clear access policies (41.5%). Administrative difficulties were also common: 40.2% of participants reported modifying clinical protocols due to medication shortages, and 48.8% observed adverse clinical consequences. Significant disparities were found according to the type of institution and level of care, as indicated by chi-square *p*-values (institution type: *p* = 0.002; level of care: *p* < 0.001), with lower medication availability in public and primary care settings. These findings indicate that access to palliative sedation medications in Ecuador is limited by structural and systemic barriers rather than economic factors alone. Improving access requires system-level interventions, including stronger regulatory frameworks, optimized supply chains, and integration of palliative care across all levels of the health system. Ensuring continuous and equitable access to essential medications is essential to reduce preventable suffering and guarantee dignified end-of-life care.

## 1. Introduction

Population aging and the increasing prevalence of non-communicable chronic diseases have significantly expanded the global need for palliative care. It is estimated that by 2060, approximately 48 million people will die each year experiencing serious health-related suffering, with more than 80% of these deaths occurring in low- and middle-income countries [1]. Although palliative care has been recognized as a human right and an essential component of universal health coverage, only a small proportion of those in need currently receive adequate services [2].

In the final stages of life, a substantial proportion of patients develop refractory symptoms, such as dyspnea, severe pain, delirium, or existential distress, that do not respond to conventional therapeutic interventions [3]. In these situations, palliative sedation may be considered when suffering is intractable and intolerable despite appropriate treatment. Palliative sedation is defined as the intentional and proportionate reduction of consciousness through sedative medications to relieve refractory suffering in terminally ill patients. This distinction is important because palliative sedation is often confused with routine symptom control; however, it should be reserved for refractory suffering and individualized in intensity and duration according to the principle of proportionality [4].

The effectiveness of this practice depends critically on the availability of essential medications, particularly benzodiazepines such as midazolam, which are widely recommended in international guidelines due to their efficacy and safety profile [3].

Despite its recognition as a public health priority, access to essential palliative care medicines remains highly unequal worldwide [5]. In low- and middle-income countries, regulatory restrictions, weak supply chains, limited institutional capacity, and gaps in professional training create structural barriers that hinder timely and equitable access to these treatments [5].

In Latin America, access to palliative care services and essential medications for symptom control continues to be uneven [6]. The *Palliative Care Atlas of the Americas 2025* highlights marked disparities between urban and rural areas, as well as limited availability of essential medicines in primary care settings: only a minority of countries report urban availability of these drugs, and even fewer have access to immediate-release oral morphine [7]. These findings reflect an insufficient and territorially unequal distribution of palliative care resources across the region. In addition, regulatory, logistical, and educational barriers reported in regional studies further restrict the appropriate prescription of opioids and sedatives, thereby exacerbating inequities in end-of-life care [8].

According to the latest official population projections from the National Institute of Statistics and Census, based on the 2022 Census and reported at mid-year, Ecuador had approximately 18 million inhabitants during the study period [9]. This provides context for the estimated national need for palliative care. The Ecuadorian health system is segmented and fragmented, with services provided by the Ministry of Public Health, the social security system, and the private sector. Medicines in public facilities and social security services are mainly financed and procured by the State or insurance-based subsystems; however, when medicines are unavailable, patients often must purchase them in private pharmacies, increasing out-of-pocket expenditure. This is particularly relevant because, in 2023, only 23.3% of the population had social insurance, and 10% had private insurance, leaving approximately two-thirds of the population dependent on the Ministry of Public Health as their main provider [9,10,11].

The Ecuadorian context intensifies these challenges. An estimated 78,000 individuals require palliative care; however, specialized services are available to approximately 0.80 per 100,000 inhabitants and are predominantly concentrated in urban areas, resulting in substantial territorial inequities. From a pharmacological perspective, opioid consumption remains extremely low at approximately 16 mg per capita, one of the lowest reported levels, and access to essential formulations, such as immediate-release oral morphine in primary care, remains restricted [12]. These limitations point to structural weaknesses in the health system’s organization, medication management, and policy implementation [13,14]. The Organic Law on Palliative Care was approved by the Ecuadorian National Assembly as a bill in 2024 and officially promulgated on 28 March 2025 [15]. Therefore, it was in effect during the data collection period, which ran from July to November, 2025. This law represents a recent regulatory advance in the national context; however, given its recent promulgation, its operational implementation, and its potential influence on physicians’ awareness of and actual access to palliative sedation medications, these aspects were not specifically assessed in this study.

These challenges are driven by interconnected factors, including regulatory barriers, inefficiencies in pharmaceutical management and distribution, and insufficient training of healthcare professionals [14]. As a result, many patients face the end of life with preventable suffering, highlighting systemic failures in the provision of palliative care.

While previous studies have documented inequalities in access to analgesics and palliative care in the region, there is a lack of evidence specifically examining the availability and access to medications used in palliative sedation from the perspective of healthcare professionals in middle-income settings, particularly in Ecuador. Understanding these barriers from the perspective of frontline providers is essential for informing evidence-based interventions and guiding health policy decision-making [16].

Therefore, this study aimed to evaluate the availability and access to medications used for palliative sedation among physicians affiliated with ASECUP and to identify the main barriers to their use in Ecuador.

## 2. Materials and Methods

### 2.1. Study Design

This study employed a quantitative, cross-sectional design that incorporated both descriptive and analytical methods. The descriptive approach assessed the availability and access to palliative sedation medications, while the analytical component examined associations between medication availability and various institutional and contextual variables. Data collection took place between 7 July and 17 November 2025.

### 2.2. Study Population

The study population comprised physicians registered with the Ecuadorian Association of Palliative Care (ASECUP) who specialized or had experience in palliative care. This group is a nationwide network of professionals with formal training and clinical expertise. Eighty-two eligible physicians represented the entire accessible population within ASECUP. As the main organized body of palliative care specialists in Ecuador, all members who met the inclusion criteria were invited using a census approach. Participants worked at all care levels and in public, private, and mixed institutions nationwide.

### 2.3. Inclusion and Exclusion Criteria

Participants were included if they were healthcare professionals affiliated with ASECUP, actively involved in palliative care practice, and experienced in the use or prescription of palliative sedation. Additionally, they had to provide informed consent and complete the survey fully. Professionals were excluded if they were inactive during data collection or submitted incomplete questionnaires.

### 2.4. Data Collection Instrument

Data were collected using a structured, self-administered online questionnaire developed specifically for this study and distributed via Google Forms. The survey consisted of 28 items mapped to eight dimensions: (1) sociodemographic and professional characteristics, (2) knowledge and training in palliative sedation, (3) availability of medications, (4) access and administrative procedures, (5) clinical experiences, (6) barriers and facilitators, (7) home-based palliative sedation, and (8) recent changes in medication access. Each survey item corresponded to a specific dimension, ensuring comprehensive coverage of the topics. Furthermore, the questionnaire included 6 dichotomous questions, 5 Likert-scale items, and 17 multiple-choice questions. The full questionnaire is provided as Supplementary Material. All variables were categorical in nature.

### 2.5. Content Validity

To assess content validity, expert judgment was utilized. Specifically, five specialists in palliative care, each with more than five years of professional experience and formal specialization in the field, independently evaluated the questionnaire. Their assessment was guided by criteria including clarity, coherence, relevance, and adequacy of each item. The degree of agreement among the experts was subsequently quantified using Aiken’s V coefficient, which yielded values ranging from 0.87 to 1.00, indicating high content validity. However, test–retest reliability and internal consistency analyses were not performed, as the instrument was developed specifically for this exploratory study and was primarily intended to assess categorical dimensions of medication availability, access, and perceived barriers. Based on these findings, the instrument was deemed appropriate for assessing the intended dimensions in this exploratory study.

### 2.6. Data Collection Procedure

The survey was distributed electronically to all eligible ASECUP members via institutional communication channels. Participation was voluntary, and informed consent was obtained before completing the questionnaire. Following submission, responses were collected anonymously to ensure confidentiality. The data collection process was conducted over a predefined period, and responses were automatically recorded in a secure database. Subsequently, the dataset was reviewed to identify and address incomplete or inconsistent entries before being prepared for statistical analysis.

### 2.7. Statistical Analysis

Data were exported and analyzed with IBM SPSS Statistics version 29. Descriptive statistics, using absolute and relative frequencies, summarized all categorical variables. For the analytical component, bivariate analyses were conducted to assess associations between medication availability and key independent variables, including institution type (public, private, mixed), level of care (primary, secondary, tertiary), and geographic setting (urban vs. rural). The chi-square (χ^2^) test was used, and statistical significance was established at *p* < 0.05. Cramér’s V was used to measure the effect size for significant associations. Adjusted residuals identified which categories contributed to significance when appropriate. Because of the small sample size and the presence of categorical variables, multivariable analysis was not performed. The results provide exploratory evidence of structural patterns influencing medication availability across healthcare settings.

### 2.8. Ethical Considerations

This study received Ethics Committee approval from Universidad Técnica Particular de Loja (2025-03-INT-EO-RM-002). All participants gave informed consent. Data were collected anonymously to ensure confidentiality and ethical compliance.

### 2.9. Use of Generative Artificial Intelligence

Generative artificial intelligence tools were used exclusively for language editing and stylistic clarity. No AI tools were used for study design, data collection, statistical analysis, interpretation of results, or generation of scientific content.

## 3. Results

A total of 82 eligible physicians affiliated with ASECUP were contacted and invited to participate in the study. All 82 professionals completed the questionnaire, resulting in a 100% completion rate. All participants were physicians with formal training or clinical expertise in palliative care and were members of ASECUP.

For variables derived from multiple-response items, percentages may not sum to 100% because participants may select more than one option. For single-response items, percentages were calculated using the total number of valid responses (Table 1).

Among the participants were palliative care specialists (81.7%), followed by those in internal medicine and family medicine (7.3% each). Regarding professional experience in palliative care, 9.8% had less than 1 year, 41.5% had 1–3 years, 18.3% had 4–6 years, and 30.5% had more than 6 years. Most participants worked in urban areas (95.1%), primarily in private institutions (50.0%), and at the tertiary level of care (62.2%). Overall, the sample was predominantly composed of palliative care specialists working in urban and tertiary care settings.

Midazolam was used for palliative sedation by 82.0% of professionals, making it the most frequently utilized medication in this context. In contrast, morphine usage was reported by only 11.0% of participants (see Table 2), indicating a substantial difference in preference between these two drugs. Although more than half of the professionals (54.9%) reported constant availability of medications, a substantial proportion reported inconsistent access: 21.9% reported frequent availability and 18.3% only occasional availability. This variability highlights persistent gaps in the continuity of medication supply. The main causes of limited availability were regulatory restrictions (53.7%) and distribution problems (36.6%), while only 9.8% cited economic factors. Systemic and administrative barriers thus outweigh cost constraints.

As shown in Table 3, administrative barriers were common but varied. The most problematic were lack of stock (35.4%), extra requirements for controlled medications (25.6%), and exhaustive documentation (20.7%). When considering overall barriers, the absence of clear access policies was the most frequently identified limitation (41.5%), followed by excessive administrative requirements (28.0%) and distribution challenges (26.8%). These outcomes indicate that access barriers are primarily structural instead of purely logistical.

Despite over half of participants perceiving access as “very accessible,” a relevant proportion reported limitations in practice. Notably, 40.2% of professionals reported modifying clinical protocols due to medication shortages, and 48.8% reported observing adverse clinical consequences associated with limited availability. As a result, these findings suggest that even moderate disruptions in access can directly affect clinical decision-making, potentially leading to delays in care or changes in treatment plans, and consequently impacting patient outcomes (Table 4).

The participants identified several key strategies to improve access, including continuous supply programs (35.4%), staff training (32.9%), and increased institutional support (31.7%). Regarding recent trends, most respondents (51.2%) perceived no significant changes in access to medications, while smaller proportions reported improvements (25.6%) or deterioration (23.2%), indicating stagnation in system-level progress (Table 5).

A statistically significant association was identified between medication availability and type of healthcare institution (*p* = 0.002). Higher availability was reported in private institutions (58.7%), followed by mixed institutions (30.1%), whereas public institutions showed markedly lower availability (11.1%). These findings indicate a clear institutional disparity, with limited access disproportionately affecting the public sector (Table 6).

A strong association was also observed between the level of care and medication availability (Table 7, *p* < 0.001). Tertiary care institutions reported the highest availability (76.2%), while primary care centers showed significantly lower levels (6.3%). This disparity indicates that access to palliative sedation medications is concentrated in more complex settings, which could limit access for patients experiencing severe suffering at home.

No statistically significant association was found between geographic area (urban vs. rural) and perceived barriers to access (*p* = 0.96). Similar patterns of barriers were observed across both settings. However, this result should be interpreted with caution due to the limited representation of rural participants (Table 8).

## 4. Discussion

The results of this study provide evidence on the availability and access to medications for palliative sedation from the perspective of professionals affiliated with the Ecuadorian Association of Palliative Care, reflecting a problem that extends beyond clinical practice. The findings reveal a multifactorial issue involving regulatory, administrative, logistical, and training-related limitations, consistent with barriers reported internationally in low- and middle-income countries [17,18,19,20].

Similar barriers have been reported internationally, including regulatory complexity, limited medication availability, insufficient training, and poor guideline implementation [21]. Therefore, the barriers identified in Ecuador should not be interpreted as isolated problems, but as part of broader systemic and regulatory challenges that affect access to appropriate palliative sedation medications across different health systems. To address these issues, stakeholders should strengthen governance, establish clear protocols, and develop reliable mechanisms for medication supply to ensure equitable access to end-of-life symptom control.

Midazolam was the most frequently used medication, consistent with international practice for managing refractory symptoms such as delirium and dyspnea at the end of life [22,23,24]. However, its inconsistent availability highlights a gap between evidence-based practice and health system capacity. Previous studies support these findings in various contexts. In Ecuador, Ref. [25] reported unequal access to opioid analgesics in six cancer centers, particularly among low-income patients, those treated in primary care, and people living in remote areas. In Latin America, economic, institutional, sociocultural, and rural access barriers to palliative care have been identified among low-income patients [26]. Recent legal analyses in Ecuador also emphasize the need for clearer regulatory frameworks and universal access to quality palliative care at the end of life [27]. In Italy, palliative sedation in home care is affected by caregiver agreement, logistical factors, and medication supply [28]. Taken together, these studies indicate that access to palliative sedation medications is conditioned by interrelated barriers of a clinical, organizational, regulatory, socioeconomic, and supply nature.

Previous studies indicate that logistical constraints, medication supply issues, organizational factors, and legal restrictions directly affect the timing and feasibility of palliative sedation, particularly in resource-limited settings, and even intermittent shortages may lead to protocol modifications and suboptimal symptom control [25,26,27,28].

A central finding of this study is the institutional disparity in medication availability, with private institutions reporting better access than public ones. This suggests that, in Ecuador, timely relief of refractory suffering may depend on where patients receive care, raising important equity and ethical concerns. This pattern is consistent with international evidence showing that access to essential palliative care medicines remains limited in many settings, particularly in low- and middle-income countries, where regulatory restrictions, fragmented policies, weak implementation, and supply-chain limitations hinder equitable access [5,27,28,29]. Therefore, institutional differences in medication availability should be understood not only as a local supply problem, but also as a reflection of broader structural barriers affecting palliative care delivery.

The association between medication availability and level of care highlights significant inequities, with greater access concentrated in tertiary settings. This contradicts global recommendations advocating for the integration of palliative care, including access to essential medications, at all levels, particularly in primary care, to ensure universal health coverage [30]. Limited access at the primary level may delay symptom control and restrict timely care, especially in underserved areas [30]. Evidence shows that policy frameworks play a critical role: restrictive regulations can reduce service provision even in high-income systems, whereas clear policies, adequate funding, and effective implementation may improve equitable access and continuity of care [16,31]. In Ecuador, the lack of consolidated policies likely contributes to the observed disparities in access to palliative sedation.

The identified barriers, unclear protocols, regulatory restrictions for controlled medicines, administrative burden, and distribution challenges reflect a gap between palliative care policies and their implementation in clinical practice. Here, “policies” refers to national and institutional frameworks for palliative care integration, access to essential medicines, procurement and stock management, and prescribing procedures for controlled medications. Although international recommendations emphasize universal access to palliative care and rapid availability of essential medicines in health facilities, these principles are not always translated into clear operational procedures or reliable supply mechanisms [32]. Although Ecuador has made recent legislative advances, these have not yet translated into consistent access at the point of care, a challenge widely described in global palliative care systems [7,16,26,33]. Administrative difficulties were frequently reported and are recognized internationally as a persistent obstacle to access [34]. While many participants did not perceive major supply problems, medication availability was still identified as a key barrier. This apparent contradiction may reflect temporal fluctuations and an unequal distribution within the health system, which differs from prior studies in Ecuador and the region that report persistent shortages of essential medications, including opioids [33,35,36,37].

This finding may be explained by three non-mutually exclusive processes. First, “access” does not have a single definition: some studies measure availability or potential proximity, whereas others capture actual utilization, travel burden, acceptability, or service accommodation [38,39,40]. Second, accessibility is temporally variable and may depend on the specific time of observation, service operating conditions, transportation availability, or supply fluctuations; therefore, perceptions reported at the time of the survey may differ from previous clinical experiences involving shortages or delayed access [41,42]. Third, sustained exposure to scarcity may lead to cognitive and behavioral adaptation, including normalization of constraints, reprioritization of needs, and reduced translation of clinical need into observable demand, even when objective barriers persist [43,44,45]. Thus, high perceived accessibility does not necessarily imply reliable, timely, and continuous access to palliative sedation medications in clinical practice.

From a clinical perspective, palliative sedation is a time-sensitive intervention, and limited or delayed access to medications can prolong suffering, generate ethical challenges, and compromise the quality of end-of-life care. Ensuring the continuous availability of essential drugs is therefore a clinical imperative to guarantee adequate symptom control and dignity [46]. Although medication cost was perceived as a less relevant barrier in this study, international evidence shows that medicine affordability remains a key determinant of access, particularly where public-sector availability is limited and private-sector prices are often unaffordable [47,48]. This difference may reflect the Ecuadorian context, where regulatory and logistical barriers appear to outweigh direct costs; however, socioeconomic status remains an important limiting factor.

From a policy perspective, these findings underscore the need for targeted system-level interventions, including clear national protocols, standardized minimum medication stocks across all levels of care, simplified administrative processes, and strengthened supply chains. Integrating palliative care into primary care and expanding professional training are essential to improve equitable access and align practice with international standards [30].

No significant association was found between access barriers and geographic area; however, this finding should be interpreted with extreme caution because the rural subgroup was very small (n = 4; 4.88%). Therefore, the urban–rural analysis was underpowered, and the null result should not be interpreted as evidence of the absence of territorial differences in access to palliative sedation medications. International evidence shows that rural settings face greater difficulties in accessing palliative care, suggesting that territorial inequities may be underestimated in this study [49,50]. Overall, the findings point to a multifactorial problem involving regulatory, administrative, logistical, and economic barriers, consistent with patterns described in low- and middle-income countries [51,52,53].

Most professionals perceived no recent improvements in access, highlighting a substantial need for corrective action. Supply programs, staff training, and institutional support were identified as key strategies, consistent with international evidence showing that integrated models must combine policy implementation, logistics, and workforce education to strengthen palliative care access and quality [54,55,56,57].

**Figure 1:**
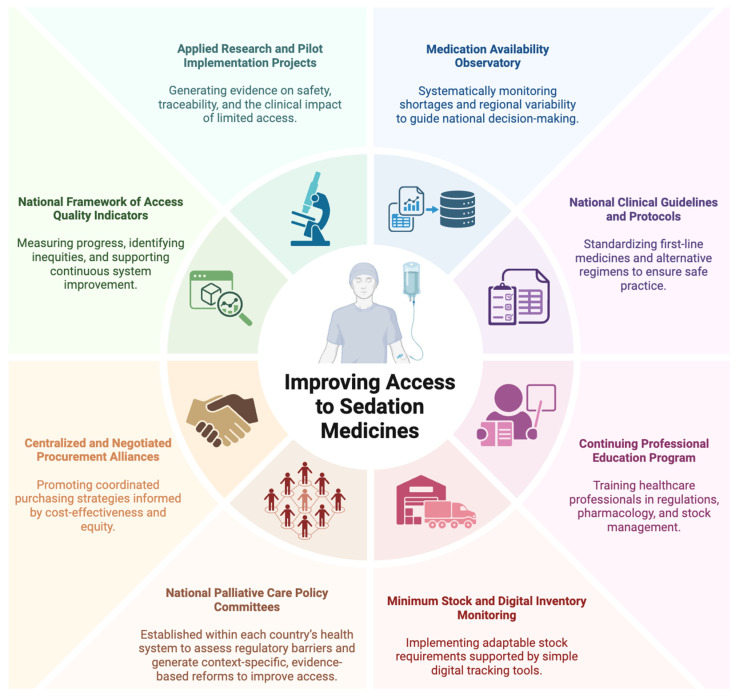
Proposed framework for action by scientific and professional palliative care associations to improve equitable access to palliative sedation medications.

Based on these findings, improvement strategies should focus on system-level interventions rather than isolated clinical actions. Scientific and professional palliative care associations can play a strategic role in translating evidence into practice by supporting medication shortage monitoring, national guidelines and standardized protocols, minimum stock recommendations by level of care, continuing education, interinstitutional coordination, and quality indicators for medication access (Figure 1). This integrated approach may strengthen governance, reduce institutional variability, and promote equitable access to palliative sedation medications in health systems facing similar structural barriers.

Although the sample size of 82 participants might initially appear small, it is crucial to emphasize that this cohort represents a complete census of the accessible population of formal palliative care specialists affiliated with ASECUP, the principal organized network for these professionals in Ecuador. Consequently, this sample size is methodologically robust and highly representative of the specialized workforce within this specific national context. Nevertheless, this study has limitations. Because the sample consisted exclusively of ASECUP-affiliated physicians, the generalizability of the findings to non-specialized practitioners may be limited. Requiring prior experience in the use or prescription of palliative sedation may have introduced selection bias, as physicians without such experience were not included. Therefore, the magnitude of access barriers may have been underestimated. Additionally, data were self-reported and subject to potential perception bias, and rural representation was limited. Although the questionnaire showed high content validity based on expert judgment and Aiken’s V, test–retest reliability and internal consistency analyses for the Likert-scale items were not conducted. Therefore, future studies should further assess the instrument’s psychometric properties, including temporal stability and internal consistency, before broader application in other populations or settings. Furthermore, the cross-sectional design precludes causal inferences. To advance understanding of access dynamics, future research should employ mixed methods, integrate qualitative perspectives, and objectively assess medication supply systems.

Despite these limitations, this study provides one of the first national analyses focused on medications for palliative sedation in Ecuador. It contributes to the field by demonstrating that access barriers are not isolated but systemic, and that improving access requires coordinated clinical, institutional, and policy-level interventions. Ensuring equitable and continuous access to essential medications is fundamental to reducing preventable suffering and advancing dignified end-of-life care.

## 5. Conclusions

This study shows that access to medications for palliative sedation in Ecuador is inconsistent and unequal, mainly due to regulatory, administrative, and supply-related barriers. These limitations affect clinical practice by forcing protocol modifications and may compromise timely relief of refractory suffering at the end of life.

By providing national evidence from palliative care physicians, this study helps close an important knowledge gap in middle-income settings. Its findings can inform future research, support the development of national guidelines, strengthen medication supply systems, and guide policies aimed at equitable access to palliative sedation.

Scientific and professional associations may play a key role in translating these findings into practice by monitoring access, providing professional training, developing protocols, and establishing quality indicators. Ensuring continuous access to essential medications is fundamental to reducing preventable suffering and protecting dignity at the end of life.

## Figures and Tables

**Table 1:** Characteristics of ASECUP professionals.

Variable	Frequency	%
**Specialty**		
Palliative medicine	67	81.71
Internal medicine	6	7.32
Anesthesiology	3	3.66
Family medicine	6	7.32
**Experience in palliative care**		
Less than 1 year	8	9.76
1–3 years	34	41.46
4–6 years	15	18.29
More than 6 years	25	30.49
**Work area**		
Urban area	78	95.12
Rural area	4	4.88
**Type of institution**		
Public	15	18.29
Private	41	50.00
Mixed	26	31.71
**Level of care of the institution**		
Primary	11	13.41
Secondary	20	24.39
Tertiary	51	62.20

(Source: Authors’ questionnaire, 2025).

**Table 2:** Availability and access to palliative sedation medications.

Variable	Frequency	%	95% CI
**Most frequently used medications**			
Midazolam	73	82.02	80.93–94.43
Morphine	9	10.98	5.57–19.07
**Routine availability**			
Always	45	54.88	44.09–65.32
Frequently	18	21.95	14.06–31.78
Occasionally	15	18.29	11.10–27.68
Rarely	3	3.66	1.04–9.44
Never	1	1.22	0.13–5.66
**Cause of medication shortages**			
Distribution problems	30	36.59	26.77–47.33
High costs	8	9.76	4.72–17.56
Strict regulation	44	53.66	42.90–64.17

(Source: Authors’ questionnaire, 2025). Note: Percentages may not add up to 100% because this table includes multiple-response items. Participants could select more than one option.

**Table 3:** Barriers to access to palliative sedation medications.

Variable	Frequency	%	95% CI
**Frequency of administrative problems**			
Never	18	21.95	14.06–31.78
Rarely	22	26.83	18.16–37.10
Sometimes	24	29.27	20.26–39.71
Frequently	13	15.85	9.19–24.88
Always	5	6.10	2.36–12.85
**Problematic procedures**			
Extensive documentation	17	20.73	13.06–30.43
Prolonged review and authorization	15	18.29	11.10–27.68
Lack of stock	29	35.37	25.67–46.08
Additional requirements for controlled medications	21	25.61	17.12–35.79
**Supply problems**			
Yes	22	26.83	18.15–37.10
No	52	63.41	52.67–73.23
Not sure	8	9.76	4.72–17.56
**Main barrier**			
High costs	3	3.66	1.04–9.44
Distribution problems	22	26.83	18.15–37.10
Excessive administrative requirements	23	28.05	19.20–40.40
Lack of clear access policies	34	41.46	31.24–52.26

(Source: Authors’ questionnaire, 2025) Note: Percentages may not add up to 100% because this table includes multiple-response items. Participants could select more than one option.

**Table 4:** Perceptions and experiences regarding access to medications.

Variable	Frequency	%	95% CI
**Accessibility of palliative sedation**			
Very accessible	45	54.88	44.09–65.32
Moderately accessible	21	25.61	17.12–35.79
Poorly accessible	14	17.07	10.14–26.29
Inaccessible	2	2.44	0.51–7.59
**Adjustment of protocols due to medication shortages**			
Yes	33	40.24	30.12–51.04
No	49	59.76	48.96–69.88
**Adverse clinical consequences**			
Yes	40	48.78	38.16–59.48
No	42	51.22	40.52–61.84

(Source: Authors’ questionnaire, 2025).

**Table 5:** Strategies to improve access to medications.

Variable	Frequency	%	95% CI
**Facilitating factors**			
Supply programs	29	35.37	25.01–45.73
Institutional support	26	31.71	21.62–41.80
Staff training	27	32.93	22.82–43.04
**Recent changes**			
Improved	21	25.61	16.15–35.07
Worsened	19	23.17	14.04–32.30
No changes	42	51.22	40.38–62.06

(Source: Authors’ questionnaire, 2025). Note: Percentages may not add up to 100% because this table includes multiple-response items. Participants could select more than one option.

**Table 6:** Availability and type of institution.

	Public (%)	Private (%)	Mixed (%)	Total (%)	*p*-Value
**Availability of medications**					
Very frequent	11.11	58.73	30.15	100.00	0.002
Infrequent	42.10	21.05	36.84	100.00	0.002
**Total**	18.29	50.00	31.70	100.00	

(Source: Authors’ questionnaire, 2025). Note: These tables report chi-square *p*-values for association analyses; 95% confidence intervals were not included because prevalence proportions were not estimated.

**Table 7:** Availability of medications according to the level of care of the institution.

	Primary (%)	Secondary (%)	Tertiary (%)	Total (%)	*p*-Value
**Availability of medications**					
Very frequent	6.34	17.46	76.19	100.00	0.001
Infrequent	36.84	47.36	15.78	100.00	0.001
**Total**	13.41	24.39	62.19	100.00	

(Source: Authors’ questionnaire, 2025). Note: These tables report chi-square *p*-values for association analyses; 95% confidence intervals were not included because prevalence proportions were not estimated.

**Table 8:** Work setting and main barriers to access medications.

	High Costs (%)	Distribution Problems (%)	Administrative Requirements (%)	Unclear Policies (%)	Total (%)	*p*-Value
**Work setting**						
Urban	3.84	26.92	28.20	41.02	100.00	0.96
Rural		25.00	25.00	50.00	100.00	0.96
**Total**	3.65	26.82	28.04	41.46	100.00	

(Source: Authors’ questionnaire 2025). Note: These tables report chi-square *p*-values for association analyses; 95% confidence intervals were not included because prevalence proportions were not estimated.

## Data Availability

Data and analysis scripts relevant to this study are available from the corresponding author upon request.

## Supplementary Materials

The following supporting information can be downloaded at: https://www.mdpi.com/article/10.3390/ijerph23070901/s1, Questionnaire on Availability and Access to Medications for Palliative Sedation.

## Abbreviations

The following abbreviations are used in this manuscript:

ASECUP	Ecuadorian Association of Palliative Care
EAPC	European Association for Palliative Care
WHO	World Health Organization
