# Supplementary material for: Availability and Access to Palliative Sedation Medications in Ecuador: A National Survey of Palliative Care Physicians
Source: Int J Environ Res Public Health. 2026 Jul 14;23(7):901. doi: 10.3390/ijerph23070901 (PMC13409946; doi:10.3390/ijerph23070901)
Supplement: Supplementary file 1 [file ijerph-23-00901-s001.zip › ijerph-4367125-supplementary.pdf]

Supplementary Material

Questionnaire on Availability and Access to Medications for Palliative Sedation

Dimension 1. Demographic Data and Professional Profile

1. **Specialty**
   • Palliative medicine
   • Oncology
   • Internal medicine
   • Anesthesiology
   • Family medicine
2. **Years of experience in palliative care:**
   • Less than 1 year
   • 1–3 years
   • 4–6 years
   • More than 6 years
3. **Geographic location of your workplace**
   • Urban area
   • Rural area
4. **Type of institution where you work**
   • Public
   • Private
   • Mixed
5. **Level of palliative care provided by your institution**
   • Primary
   • Secondary
   • Tertiary

Dimension 2. Knowledge and Training in Palliative Sedation

6. **Have you received formal training in palliative sedation?**
   • Yes
   • No
7. **If you answered "Yes" to the first question in this section, how many hours of training have you received in the past year?**
   • Less than 10 hours
   • 10–20 hours
   • More than 20 hours
   • Did not answer the first question in this section
8. **If you answered "Yes" to the first question in this section, do you consider that the training received was sufficient to address palliative sedation needs in your practice?**
   • Yes
   • No
   • Did not answer the first question in this section

9. **If you answered “Yes” to the first question in this section, how often do you participate in training or continuing education activities on palliative sedation?**
   • Monthly
   • Every 3 months
   • Every 6 months
   • Annually
   • Never
   • Did not answer the first question in this section

Dimension 3. Medication Availability

10. **Which medications do you use most frequently for palliative sedation?**
    • Midazolam
    • Morphine
    • Propofol
11. **How often are the medications required for palliative sedation available at your workplace?**
    • Always
    • Frequently
    • Occasionally
    • Rarely
    • Never
12. **Are there supply problems with medications for sedation in your institution?**
    • Yes
    • No
    • Not sure
13. **To which factors do you attribute the lack of availability of these medications?**
    • Distribution problems
    • High costs
    • Strict control and regulatory policies

Dimension 4. Access to Medications and Procedures

14. **How often do you face administrative problems in obtaining the required medications?**
    • Never
    • Rarely
    • Sometimes
    • Frequently
    • Always
15. **Which administrative procedures are most problematic in your experience**?
    • Extensive documentation
    • Prolonged review and authorization
    • Lack of stock
    • Additional requirements for controlled medications
16. **How accessible do you consider palliative sedation to be in your institution for patients who require it?**

• Very accessible
• Moderately accessible
• Poorly accessible
• Inaccessible

Dimension 5. Practical Experiences

17. **Have you had to change or adjust the palliative sedation protocol due to a lack of medications?**
    • Yes
    • No
18. **If you answered "Yes" to the previous question, what alternatives have you used?**
    • Dose adjustment of other available medications
    • Use of less effective medications
    • Patient referral
    • Did not answer the previous question
19. **Have you observed adverse clinical consequences due to lack of access to medications for palliative sedation?**
    • Yes
    • No

Dimension 6. Barriers and Facilitators

20. **In your opinion, what is the main barrier to access to palliative sedation medications in your institution?**
    • High costs
    • Distribution problems
    • Excessive administrative requirements
    • Lack of clear access policies
21. **What factor facilitates access to these medications in your institution?**
    • Continuous supply programs
    • Institutional support
    • Staff training and education
    • Improved storage infrastructure

Dimension 7. Home-Based Sedation

22. **Do you provide home-based sedation?**
    • Yes
    • No
23. **If you answered "Yes" to the first question in this section, how often do you provide home-based palliative sedation in your professional practice?**
    • Whenever necessary
    • Occasionally
    • Never
    • Did not answer the first question in this section

24. **If you answered “Yes” to the first question in this section, do you have all the necessary medications for palliative sedation when you are at the patient’s home?**
    • Yes, always
    • Sometimes
    • Never
    • Did not answer the first question in this section
25. **If you answered “Yes” to the first question in this section, how adequate do you consider the training you have received to manage palliative sedation at home?**
    • Very adequate
    • Adequate
    • Inadequate
    • I have not received specific training
    • Did not answer the first question in this section
26. **If you answered “Yes” to the first question in this section, what level of support do you receive from your institution to provide home-based palliative sedation?**
    • Full support: medications, technical support, and consultation
    • Partial support: some resources or consultation
    • None
    • Did not answer the first question in this section
27. **If you answered “Yes” to the first question in this section, what are the main difficulties you encounter in the practice of home-based palliative sedation?**
    • Lack of specific medications
    • Difficulty with continuous monitoring
    • Limited family support
    • Did not answer the first question in this section

Dimension 8. Recent Changes

28. **Have you observed any recent changes in access to these medications over the past year?**
    • Yes, access has improved
    • Yes, access has worsened
    • There have been no changes
